# In Adult Skeletal Muscles, the Co-Receptors of Canonical Wnt Signaling, Lrp5 and Lrp6, Determine the Distribution and Size of Fiber Types, and Structure and Function of Neuromuscular Junctions

**DOI:** 10.3390/cells11243968

**Published:** 2022-12-08

**Authors:** Lea Gessler, Christopher Kurtek, Mira Merholz, Yongzhi Jian, Said Hashemolhosseini

**Affiliations:** 1 Institute of Biochemistry, Medical Faculty, Friedrich-Alexander-University of Erlangen-Nürnberg, 91054 Erlangen, Germany; 2 Muscle Research Center, Friedrich-Alexander-University of Erlangen-Nürnberg, 91054 Erlangen, Germany

**Keywords:** Lrp5, Lrp6, canonical Wnt signaling, synaptic gene expression, neuromuscular junction

## Abstract

Canonical Wnt signaling is involved in skeletal muscle cell biology. The exact way in which this pathway exerts its contribution to myogenesis or neuromuscular junctions (NMJ) is a matter of debate. Next to the common co-receptors of canonical Wnt signaling, Lrp5 and Lrp6, the receptor tyrosine kinase MuSK was reported to bind at NMJs WNT glycoproteins by its extracellular cysteine-rich domain. Previously, we reported canonical Wnt signaling being active in fast muscle fiber types. Here, we used conditional Lrp5 or Lrp6 knockout mice to investigate the role of these receptors in muscle cells. Conditional double knockout mice died around E13 likely due to ectopic expression of the Cre recombinase. Phenotypes of single conditional knockout mice point to a very divergent role for the two receptors. First, muscle fiber type distribution and size were changed. Second, canonical Wnt signaling reporter mice suggested less signaling activity in the absence of Lrps. Third, expression of several myogenic marker genes was changed. Fourth, NMJs were of fragmented phenotype. Fifth, recordings revealed impaired neuromuscular transmission. In sum, our data show fundamental differences in absence of each of the Lrp co-receptors and suggest a differentiated view of canonical Wnt signaling pathway involvement in adult skeletal muscle cells.

## 1. Introduction

In vertebrates, WNT glycoproteins are a family of signaling molecules also involved in myogenesis and, through both canonical and non-canonical Wnt pathways, regulate muscle formation and maintenance of adult tissue homeostasis [1]. Canonical Wnt signaling is initiated by WNT glycoproteins which bind to Frizzled (FZD) and low-density lipoprotein receptor-related protein (Lrp) receptor complex, thereby leading to the inactivation of glycogen synthase kinase 3β (GSK3β) through dishevelled (DSH). In the absence of WNT stimulation, β-catenin forms a destruction complex with adenomatosis polyposis coli (APC), AXIN1/AXIN2 and GSK3β [2]. Phosphorylation of β-catenin by CK1 and GSK3β causes ubiquitination and proteasome-mediated degradation of β-catenin. WNT stimulation results in the activation of DSH, which leads to phosphorylation-dependent recruitment of AXIN1/AXIN2 to the Lrp5/6 receptors and disassembly of the β-catenin destruction complex. Stabilized β-catenin accumulates in the cytoplasm and translocates to the nucleus. There, it complexes with T cell factor/lymphoid enhancer factor (TCF/LEF) transcription factors and acts as a transcriptional coactivator to induce the context-dependent expression of WNT/β-catenin target genes [3].

In vertebrates, there are several FZD receptors, but only two co-receptors (Lrp5, Lrp6) involved in canonical Wnt signaling [4]. Lrp5 and Lrp6 are highly homologous transmembrane receptors, widely co-expressed in embryonic and adult tissues, and crucial in the initiation of Wnt signaling [5,6,7,8]. Active Wnt signaling requires binding of WNT to FZD and Lrp5/Lrp6 thereby clustering these receptors, resulting in the formation of ternary complexes [9,10]. However, Lrp5 and Lrp6 also exhibit distinct characteristics. First, in frog embryos Lrp6 alone is sufficient to induce axis duplication, while Lrp5 is not [5]; second, WNT ligands exhibit a preferential use of Lrp5 or Lrp6 [11]; third, several reports suggest that Lrp6 displays broad actions that go beyond Wnt signal transduction [11,12]. Lrp6 can interact with multiple protein partners and acts as a co-receptor for many other extracellular cues [13,14,15].

AXIN2 is a direct target of TCF/LEF-mediated transcription [16,17,18]. AXIN2 is expressed in many cells where Wnt signaling activity was detected [19] and is widely used as a reporter for canonical Wnt signaling activity. Gene transcription mediated by the transcriptional co-activator β-catenin is an important regulator of muscle cell differentiation [20,21]. Previously, our lab demonstrated that AXIN2 is expressed in adult skeletal muscle fibers and canonical WNT proteins concomitantly stimulate both canonical Wnt signaling as well as Axin2 expression during muscle cell differentiation to regulate myotube formation [22].

The Hippo pathway is an evolutionarily conserved signaling cascade that regulates organ size and tissue homeostasis [23]. There are two key downstream transcriptional co-activators described, yes-associated protein (YAP1 or YAP) and transcriptional co-activator with PDZ-binding motif (TAZ). It was reported that in the absence of WNTs, YAP and TAZ are components of the β-catenin destruction complex, and YAP/TAZ are associated with the destruction complex by binding to AXIN [24]. Interestingly, YAP/TAZ and Lrp6 apparently compete for binding to the same domain of AXIN [24]. When cells overexpress Lrp6 or are stimulated by WNT, Lrp6 binds to AXIN1 and liberates YAP/TAZ from the destruction complex, assuming that they then induce the expression of YAP/TAZ target genes [24]. This hypothesis is supported by showing that Dickkopf 3 (DKK3), an antagonist of canonical Wnt signaling, stabilizes the cell-surface levels of Lrp6, thereby Lrp6 is uncoupled from cellular machineries, resulting in concomitant activation of β-catenin and YAP/TAZ [25]. Lrp5 variants lacking the extracellular domain act as constitutive active mutants that bind AXIN and induce LEF-1 activation by destabilizing AXIN and stabilizing β-catenin [26]. Intriguingly, overexpression of full-length Lrp5 alone has no effect on the canonical Wnt signaling but acts synergistically with WNT [26]. Accordingly, Lrp5 is shown to be associated with AXIN, and this interaction is further strengthened by WNT3a [27]. However, either full-length Lrp5 or Lrp5 variants have no effect on YAP/TAZ activity [6].

At the neuromuscular junction (NMJ), several signaling pathways are responsible for ensuring clustering of nicotinic acetylcholine receptors (AChRs) at the postsynaptic apparatus [28]. A neural isoform of a large heparan sulfate proteoglycan, called AGRIN, is released by the nerve ending and involved in both stabilization of clusters of existing acetylcholine receptors and stimulation of synaptic gene expression. To this end, AGRIN interacts with its receptor Lrp4 and thereby activates the co-receptor MuSK, a muscle-specific receptor tyrosine kinase. The clustering of AChRs serves as a hallmark for the presence of the postsynaptic apparatus within the endplate zone, the central part of each muscle fiber [28].

Muscular β-catenin gain-of-function phenotype is associated with presynaptic defects in vivo resulting from changed neuromuscular retrograde signaling [29,30,31]; however, β-catenin loss of function also affects AChR cluster size and distribution [29]. In cultured muscle cells, β-catenin exerts both positive and negative regulation of AChR clustering by either acting cytosolic as a link between RAPSN, a peripheral membrane protein required for AChR clustering at NMJs and the cytoskeleton, or negatively regulating *Rapsn* expression in the nucleus, respectively [32,33]. However, the downregulation of *Rapsn* transcription by β-catenin was found to be TCF independent [32]. On the other hand, β-galactosidase reporter was accumulated in synaptic nuclei in muscle fibers of AXIN2-lacZ reporter mice [22], suggesting that canonical Wnt/β-catenin signaling and TCF/LEF target gene expression are active at the NMJ. Similarly, in X-Gal-stained TCF/LEF-lacZ reporter mouse muscles, a pronounced neuromuscular X-Gal reporter activity is detectable [34].

Interestingly, MuSK contains in its extracellular region a Frizzled-like domain (cysteine-rich domain [CRD]) mediating its interaction with several WNTs, including WNT4, WNT11, and WNT9a in vitro [35,36,37]. Activation of the MuSK-Lrp4 complex regulates the prepatterning step, before muscle innervation, during which AChRs begin to aggregate in a central synaptic region of the muscle [38,39,40]. Moreover, in vivo knockdown of WNT4 and WNT11 affects muscle prepatterning and axon guidance, indicating a role for Wnt signaling [35,36,41]. Upon innervation, the MuSK-Lrp4 complex is further stimulated by neural AGRIN, which induces multiple signaling pathways leading to clustering and remodeling of aneural AChR clusters [42,43]. In addition to their role in prepatterning, WNTs have been shown to regulate AGRIN-induced AChR clustering in vitro [32,44].

Altogether, the role of Lrp5 and Lrp6 appears almost not understood in skeletal muscle cells, especially the way in which and whether WNT ligands signal by Lrp5 and Lrp6, or by MuSK CRD. The muscle-specific ablation of Lrp5 and Lrp6 using conditional mouse models might help to understand the role of canonical Wnt and its link to Hippo signaling during myogenesis, in adult myofibers and at NMJs. Here, we conditionally knocked out Lrp5, Lrp6, or both in the skeletal muscle lineage. While the individual knockouts were viable, the double knockout mice died prenatally due to ectopic expression of the Cre recombinase. We characterized both single knockout mice and identified very divergent roles for Lrp5 and Lrp6 in skeletal muscle cells.

## 2. Materials and Methods

### 2.1. Mouse Procedures and Genotyping

Mouse experiments were performed in accordance with animal welfare laws and approved by the responsible local committees (animal protection officer, Sachgebiet Tierschutzangelegenheiten, FAU Erlangen-Nürnberg, AZ: I/39/EE006 and TS-07/11) and government bodies (Regierung von Unterfranken). Floxed Lrp5 and Lrp6 mice were kindly provided by Dr. Gabriela G Loots. HSA-Cre mice were described before [45,46]. Mice were housed in cages that were maintained in a room with temperature 22 ± 1 °C and relative humidity 50–60% on a 12 h light/dark cycle. Water and food were provided ad libitum. Mouse mating and genotyping were performed as previously described [47]. Mice were genotyped by PCR analysis of ear biopsies DNA [45]. Mendelian frequencies were calculated using a simple MS EXCEL workbook [48]. Muscle force of the mice was measured with all four limbs by a Grip Strength Test Meter (Bioseb) [49]. All mice used were 4–7 month of age. No haploinsufficiency was detected in heterozygote floxed Lrp5/6 mice independent from the presence or absence of HSA-Cre. Throughout the manuscript, the term “control” is used for homozygous floxed Lrp5/6 mice without HSA-Cre, and “mutant” is used for homozygous floxed Lrp5 or Lrp6 mice with HSA-Cre.

### 2.2. RNA Extraction, Reverse Transcription, PCR

Total RNA was extracted from mouse tissues with TRIzol reagent (Thermo Fisher Scientific, Schwerte, Germany, 15596026) [45] and reverse transcribed with M-MuLV Reverse Transcriptase (New England Biolabs, Frankfurt am Main, Germany, M0253) according to the manufacturer’s instructions. cDNAs were used with mouse-specific primers (Supplementary Table S1) for quantitative PCR reactions using the PowerUp SYBR Green Master Mix (Thermo Fisher Scientific, Schwerte, A25743) and the C1000 Thermal Cycler with the CFX96 Real-Time PCR Detection System (Bio-Rad) according to the manufacturer’s instructions. After the PCR run, sizes of amplified DNA products were verified by agarose gel electrophoresis. Ct values of the genes of interest were normalized to Ct values of the internal control (Rpl8 gene) (normalized expression = 2^−ΔCT^) or additionally related to the control sample (fold change = 2^−ΔΔCT^) [50,51].

### 2.3. SDS-PAGE, Western Blot, Immunoprecipitation

For SDS-PAGE and immunoblotting, muscles were homogenized in RIPA lysis buffer with complete protease inhibitors (Sigma-Aldrich Chemie, Taufkirchen, Germany, 04693116001). Skeletal muscle homogenates were sonicated for 10 sec and centrifuged at 16,100× *g* at 4 °C for 5 min. Cleared lysate was used for immunoblotting experiments. Aliquots of muscle lysates were solubilized in Laemmli buffer (150 mM Tris, pH 6.8, 6% SDS, 30% glycerol, 0.3% bromophenol blue, 3% ß-mercaptoethanol), boiled at 95 °C, and loaded on 8% or 10% SDS-PAGE. Proteins were transferred to nitrocellulose membrane (Sigma Aldrich Chemie, Taufkirchen, Germany, Protran BA 85), blocked in 5% nonfat dry milk (Heirler Cenovis, Radolfzell am Bodensee, Germany, 3030) in PBS, 0.1% Tween20 (Carl Roth, Karlsruhe, Germany, 9127.1) for 1 h at room temperature. Immunoprecipitation was done with specific antibodies for endogenous proteins from hind limb muscle lysates. Antibodies were incubated with lysates overnight at 4 °C. The next day, protein A Sepharose beads (GE Healthcare Bio-Sciences AB, Solingen, Germany, 17061801) were added and incubation was continued for another 2 h. Afterwards, beads were washed 3 times, boiled in Laemmli buffer and loaded on SDS-PAGE gel. Primary antibodies were incubated at 1:500 dilution. The following antibodies were purchased from Cell Signaling Technology: Lrp5 (D80F2), Lrp6 (C5C7); from SantaCruz: Lrp5 sc-309267 (B9), Lrp6 sc-25317 (C10), GAPDH sc-257787. Corresponding secondary antibodies conjugated with horseradish peroxidase (Cell Signaling Technology, Leiden, The Netherlands, 7074 and 7076; 1:3000) were used for 2 h at room temperature. Protein bands were detected either by SuperSignal West Femto Maximum Sensitivity Substrate (Thermo Fisher Scientific, Schwerte, Germany, 34095) or by homemade chemiluminescence reagent composed of 50 mg Luminol (Sigma Aldrich Chemie, Taufkirchen, Germany, A-4685) in 200 mL 0.1 M Tris, pH 8.6, combined with 11 mg para-hydroxy-cumarinic acid (Sigma Aldrich Chemie, Taufkirchen, Germany, C-9008) in 10 mL DMSO. For that, 3 mL of the first solution and 40 µL of the second solution were mixed with 3 mL of PBS and 1.2 µL 30% H_2_O_2_. Western blot results were quantified by densitometric analysis using Fiji software (doi:10.1038/nmeth.2019). The background was subtracted with rolling ball radius 1.000 pixels and disabled smoothing option. Afterwards, protein bands of interest were labeled and measured. If not described specifically, values in mutant mice were expressed as relative values to control mice normalized to GAPDH and set to 1.0.

### 2.4. Histochemical Staining, Immunofluorescence Staining, Quantitative 3D Morphometrical Imaging, Color Deconvolution, X-Gal Staining, Fluorescence Microscopy

For histochemical and immunofluorescence analysis, all gastrocnemius and soleus muscles were quick-frozen in prechilled isopentane. Cryotome sectioned muscles were either used for histochemical or for immunofluorescence stainings. Sections were embedded in DPX or mowiol. Hematoxylin and eosin staining: sections were incubated 15 min in Mayer hemalum solution (Merck, Darmstadt, Germany, 109249), washed 10 min in tap water, dipped 6 times in a solution containing 96% ethanol and 4% HCl, 10 min in tap water, 1 min in 70% ethanol, 2 min in Eosin (Merck, Darmstadt, Germany, 115935), 1 min in 100% ethanol.

COX: Sections were incubated 60 min at 37 °C in a solution containing 50 mM phosphate buffer, pH 7.4, 3,3-di-aminobenzidinetetrahydrochloride (DAB; Sigma Aldrich, Taufkirchen, D8001), catalase (20 mg/mL; Sigma Aldrich, Taufkirchen, Germany, S41168), sucrose and CYCS/cytochrome c (Sigma-Aldrich, Taufkirchen, Germany, C2037). Afterwards they were washed in H_2_O and embedded. For immunofluorescence stainings, muscles were fixed in 2% PFA (paraformaldehyde), permeabilized for 15 min in 0.1% Triton X-100 and 100 mM Glycin and blocked with M.O.M blocking reagent (Vector Laboratories, Eching, Germany) for 1 h. The following antibodies were used for staining: anti-myosin heavy chain type I (BA-F8, DSHB; 1:1000) and anti-myosin heavy chain type IIa (SC-71, DSHB; 1:1000). Secondary antibody conjugated to Alexa Fluor 488 (Invitrogen, Darmstadt, Germany, A-21206; 1:1000) and Alexa Fluor 546 (Invitrogen, Darmstadt, Germany, A-21123; 1:1000) were used for detection [52].

For immunofluorescence experiments, diaphragms were dissected and fixed overnight in 4% PFA. Diaphragms were blocked in 100 mM glycine for 1 h at 4 °C; permeabilized in 0.6% Triton X-100, 5% BSA, and 1% FCS for 1–2 h, and incubated with rhodamine-α-bungarotoxin (Invitrogen, Darmstadt, Germany, BTX; 1:2500) for 1 h [49]. Stainings and endplate band width measurements were documented using a Zeiss Axio Examiner Z1 microscope (Carl Zeiss MicroImaging, Göttingen, Germany) equipped with an AxioCam MRm camera (Carl Zeiss MicroImaging, Göttingen, Germany) and ZEISS AxioVision Release 4.9 (Carl Zeiss MicroImaging, Göttingen) [49].

For quantitative 3D morphometrical imaging, mice soleus or diaphragm muscle was dissected and fixed in 2% PFA for 2 h at 4 °C. For detection of AChRs, muscle bundles containing 5–10 fibers were prepared and stained with BTX (Invitrogen, Darmstadt, Germany, BTX; 1:2500) for 1 h at room temperature. Stained bundles were washed three times for 5 min in phosphate buffered saline (PBS) and embedded in Mowiol. Then, 3D images of NMJs were taken with a 63 × 1.4 numerical aperture oil objective (Zeiss Examiner Z1, Carl Zeiss MicroImaging, Göttingen, Germany) at 55 ms exposure time. Images were deconvoluted and analyzed using different modules in AxioVision software (ZEISS AxioVision Release 4.9, Carl Zeiss MicroImaging, Göttingen, Germany). The following parameters were determined for each NMJ: volume, surface, grey sum, grey mean and number of fragments. For each genotype, more than 50 NMJs were analyzed [49]. Fluorescence grey sum depicts the total of grey values for each pixel in the acquired image, whereas fluorescence grey mean is fluorescence grey sum divided by the number of pixels in the acquired image [53].

For X-gal staining, whole soleus muscles were fixed for 1 h in PFA, 3 × 5 min washed in PBS, and incubated in X-Gal staining solution at 37 °C, consisting of 0.75 mg/mL X-Gal, 5 mM potassium ferricyanide, 5 mM potassium ferrocyanide, 0.01% sodium deoxycholate, 0.02% NP-40, 2 mM magnesium chloride and 20 mM Tris in phosphate buffered saline. Stained muscle tissues were visualized using the Zeiss Discovery V8 stereo microscope equipped with an AxioCam HRm camera and the Zeiss ZEN blue software Release 3.6 (Carl Zeiss MicroImaging, Göttingen, Germany). For quantification of blue staining, the “Color Deconvolution 2” Fiji plugin [54,55] together with Fiji was used to separate the blue image. A threshold for intensity has been attributed to the blue image, which was correlated with the amount of X-Gal staining for each image. After selecting the image of the whole muscle by the polygon button, the integrated density was measured.

### 2.5. Nerve Muscle Preparation and Electrophysiological Recordings

Diaphragm-phrenic nerve preparations were maintained ex vivo in Liley’s solution gassed with 95% O_2_, 5% CO_2_ at room temperature [56]. The recording chamber had a volume of approximately 1 mL and was perfused at a rate of 1 mL/min. The nerve was drawn up into a suction electrode for stimulation with pulses of 0.1 msec duration. The preparation was placed on the stage of a Zeiss Axio Examiner Z1 microscope (Carl Zeiss MicroImaging, Göttingen, Germany) fitted with incident light fluorescence illumination with filters for 547 nm/red (Zeiss filter set 20) fluorescing fluorophore (Carl Zeiss MicroImaging, Göttingen, Germany). The compound muscle action potential (CMAP) was recorded using a micropipette with a tip diameter of approximately 10 µm filled with bathing solution. The electrode was positioned so that the latency of the major negative peak was minimized. The electrode was then positioned 100 µm above the surface of the muscle, and CMAP was recorded. Trains of repetitive nerve stimulations (5 Hz) were performed at 2 min intervals, and the ratio of CMAP amplitudes (mean (20th–25th)/2nd) was calculated [49,57]. To block muscle action potentials so that EPPs (endplate potentials) and EPCs (endplate currents) could be recorded at 1 Hz for 100 s [58,59], µ-conotoxin GIIIB (µ-CTX, 2 µM; Peptide Institute, Osaka, Japan) was added to Lilly’s solution. EPPs were recorded at 5 Hz for 5 s and at 20 Hz for 10 s. Decrements of EPPs were calculated employing the mean of the first and the last five recordings. Concurrently, clustered AChRs at NMJs were labeled by adding 0.5 × 10^−8^ M of BTX (Life Technologies, Darmstadt, Germany) to the same Lilly solution. In some experiments, the effect of the toxin wore off after 1–2 h, and contractions resumed in response to nerve stimulation. These preparations were then exposed to the toxin for the second time. Two intracellular electrodes (resistance 10–15 MΩ) were inserted within 50 µm of the NMJs under visual inspection [53]. Current was passed through one electrode to maintain the membrane potential within 2 mV of −75 mV, while voltage transients were recorded with the other. Signals were amplified by an Axoclamp 900 A and digitized at 40 kHz by a Digidata 1440 A under the control of pCLAMP 10 (Molecular Devices, Sunny Vale, CA, USA). Voltage records were filtered at 3 kHz and current records at 1 kHz (8-pole Bessel filter). Current transients were recorded using the two-electrode voltage-clamp facility of the Axoclamp 900 A. Clamp gains were usually 300–1000, reducing the voltage transients to <3% of their unclamped amplitudes. At most NMJs, 50–100 spontaneous quantal events were recorded during a period of 1 min. Records were analyzed using pCLAMP 10. Spontaneous events were extracted using the “template search” facility and edited by eye to remove obvious artifacts. Events recorded from each NMJ were averaged, and the amplitude and frequency were determined [49].

### 2.6. Statistical Analysis

Statistical analysis was performed in GraphPad Prism 9 (GraphPad software, San Diego, USA) as indicated. Data are presented as mean values, and the error bars indicate S.D. The number of biological replicates per experimental variable (n) is usually *n* > 5 or as indicated in the figure legends. For all data with mice, a minimum of 3 mice were studied. The significance was calculated by unpaired 2-tailed student *t* test, or as indicated by the figure legends, and provided as real *p* values that are believed to be categorized for different significance levels, **** *p* < 0.0001, *** *p* < 0.001, ** *p* < 0.01, or * *p* < 0.05.

## 3. Results

### 3.1. Differential Impairment of Viability and Body Weight in Conditional Skeletal Muscle-Specific Lrp5, Lrp6, or Double Knockout Mice

**Figure 1:**
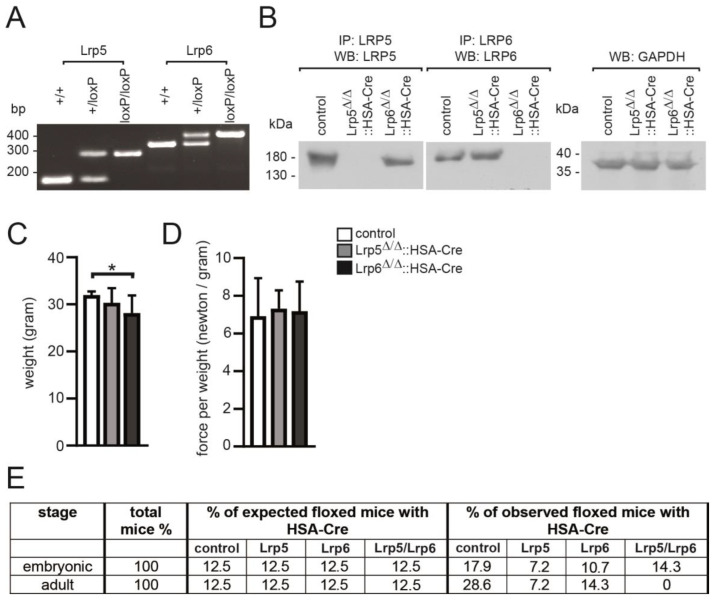
Phenotype of conditional Lrp5, Lrp6, and double knockout mice. (**A**) Agarosegel image of PCR genotyping demonstrates the heterozygous and homozygous floxed alleles of Lrp5 and Lrp6. (**B**) Images of X-ray exposure of Western blot membrane of muscle lysates (GAPDH) or immunoprecipitations of Lrp5 or Lrp6 using muscle hind limb lysates of control and mutant mice as indicated. (**C**) The graph reflects the body weight of control and single knockout mice at adulthood (4–7 months of age). Note that in comparison with controls, body weight of conditional Lrp6 knockouts is slightly reduced (*n* = 8–16 mice per genotype). (**D**) Muscle force is presented by a graph summarizing force per weight for control and conditional single knockout mice. (**E**) Distribution of offspring of conditional Lrp5 and Lrp6 knockout mice (*n* = 8–16 mice per genotype). Note that while conditional single knockout mice are born in agreement with mendelian distribution, double knockout die at embryonic stage. All labeling of graph bars represents genotype of mice as indicated in (**D**). * *p* < 0.05.

The overall topology of the canonical Wnt co-receptors Lrp5 and Lrp6 is very similar and they are believed to be mandatory for induction of canonical Wnt signaling, but their Wnt signaling capabilities are reported being not equivalent [6]. To study their role regarding canonical Wnt signaling in adult muscle fibers and at NMJs, we generated conditional Lrp5 and Lrp6 knockout mice by breeding them with HSA-Cre mice, which express Cre recombinase under the control of the human skeletal actin (HSA) promoter [60]. We used two previously described conditional floxed alleles for Lrp5 and Lrp6 [61]. PCR-based genotyping analysis ascertained identification of heterozygous and homozygous floxed alleles (Figure 1A). Accordingly, Lrp5 and Lrp6 protein amounts were significantly reduced in hind limb muscle lysates of conditional Lrp5 or Lrp6 knockout mice (Figure 1B). Note that not all offspring genotypes followed mendelian distribution (Figure 1E). While individual single and double mutant mice were detectable at early embryonic stage, double knockout mice died around embryonic day E13. It appears rather unlikely that mid-embryonal death of conditional double knockout mice is related to a skeletal muscle phenotype, but the observed lethality might be related to previously reported ectopic expression of the Cre recombinase [60] and demands to be investigated using different muscle-specific Cre mice. Here, the analysis of the double knockout is beyond the scope of this manuscript. We focused on characterization of the conditional single knockout mice in adult skeletal muscle fibers. At adulthood, body weight of Lrp6 knockout mice was slightly, but significantly, lower in comparison with control mice (Figure 1C). Muscle strength of individual Lrp5 or Lrp6 knockout mice appeared not to be different in comparison with control mice (Figure 1D). In total, these data demonstrate little significant reduction of body weight in conditional Lrp6 knockout mice, and mid-embryonal lethality of double knockout mice.

### 3.2. Oxidative Metabolism Is Reduced in the Skeletal Muscles of Conditional Lrp5 or Lrp6 Knockout Mice

**Figure 2:**
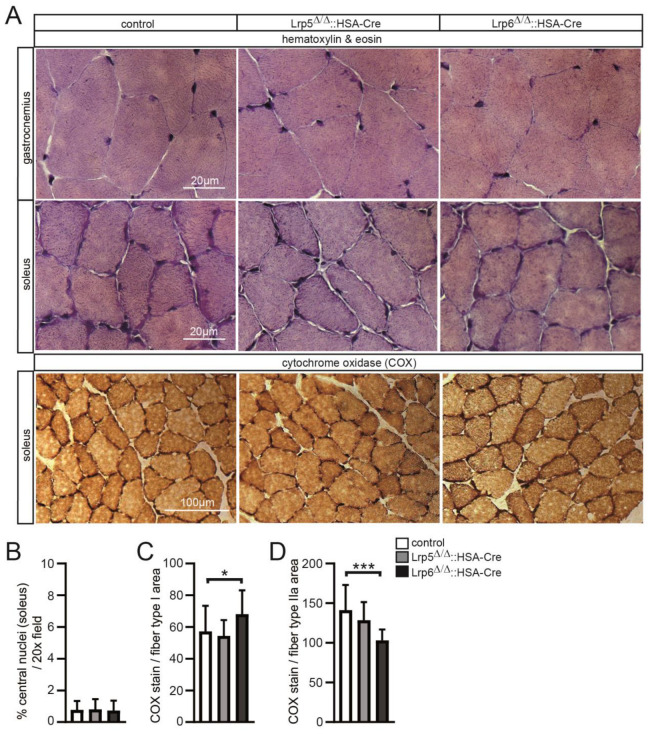
Reduced oxidative metabolism in type IIa fibers of conditional Lrp5 or Lrp6 knockout muscles. (**A**) Upper part, hematoxylin and eosin stain of hind limb muscle cross sections of control and conditional Lrp5 or Lrp6 knockout mice did not reveal any apparent changes. Lower part, Cox stain of soleus muscles with same genotypes as depicted in (**A**). (**B**) Graph presents number of central nuclei in control and mutant hind limb muscles. (**C**,**D**) Graphs summarize quantification of Cox stains (**A**) after color deconvolution by Fiji. Per image (20× field), 15 dark-colored fibers, representing oxidative metabolism, or 15 less-colored fibers, representing glycolytic metabolism, were analyzed. For color deconvolution, DAB filter was used and a threshold of 0 to 50 generated a grey scale image. Raw integrated density was measured and normalized by total fiber area. All labeling of graph bars represents genotype of mice as indicated in (**D**). *** *p* < 0.001, * *p* < 0.05.

Canonical Wnt signaling was identified in type IIa and IIx adult muscle fibers and being linked to muscle fiber cross-sectional area [22]. To obtain a first impression of any impairments of muscle cross section histology in conditional Lrp5 or Lrp6 knockout mice, we performed typical hematoxylin and eosin staining using hind limb muscles, gastrocnemius and soleus (Figure 2A). No apparent difference of histology was detectable between control and conditional Lrp5 or Lrp6 knockout mice (Figure 2A). We also did not observe any signs of myopathy in the individual conditional knockout mice, as the number of fibers with centrally located nuclei or apoptotic cells was similar in the muscles of control and conditional knockout mice (Figure 2B; data not shown). Cytochrome oxidase (COX) histochemical staining of muscle cross sections typically labels fibers with high mitochondrial content, such as slow fiber types (type I) or fast oxidative type fibers (type IIa), with a darker color. At first glance, we did not observe any differences visually inspecting COX-stained hind limb cross sections (Figure 2A). We employed color deconvolution using Fiji software to quantify COX staining. Interestingly, more COX staining was detected in conditional Lrp6 knockout muscle fibers type I (Figure 2C) and gradually less COX activity was quantified in bright-colored fibers of soleus muscle, most likely type IIa fibers, of conditional Lrp6 knockout mice in comparison with controls (Figure 2D).

### 3.3. The Numbers and Cross-Sectional Areas of Skeletal Muscle Fibers Are Altered in Conditional Lrp5 or Lrp6 Knockout Mice

**Figure 3:**
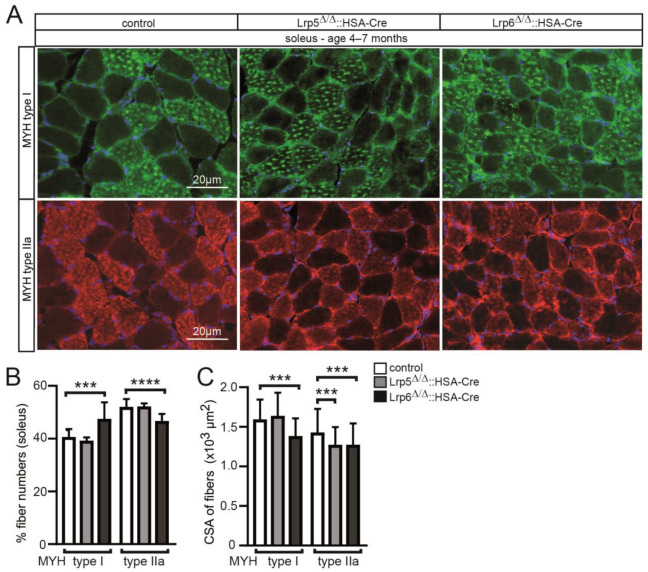
Muscle fiber type distribution and size are changed in conditional Lrp5 or Lrp6 knockout mice. (**A**) Soleus muscle of adult control and conditional Lrp5 or Lrp6 knockout mice were cross-sectioned and immunostained with myosin heavy chain antibodies. Representative images are shown. (**B**) Number of fibers of type I and IIa was counted and is presented by a graph. Note that conditional Lrp6 knockout muscles are composed of slightly more type I and less type IIa fibers. (**C**) Cross-sectional areas of type I and type IIa muscle fibers of control and Lrp5 or Lrp6 conditional knockout mice are summarized. Note that cross-sectional areas are differently reduced in Lrp5 or Lrp6 mutant mice in comparison with controls. All labeling of graph bars represents genotype of mice as indicated in (**C**). **** *p* < 0.0001, *** *p* < 0.001.

Previously, our lab detected active canonical Wnt signaling in type IIa and type IIx muscle fibers of the hind limbs and in diaphragms [22]. We asked whether the same fiber types are impaired in conditional Lrp5 or Lrp6 knockout mice. Using cross sections and immunofluorescence stainings with myosin heavy chain-specific antibodies of hind limb muscles of the control and mutant mice several differences were detected (Figure 3). Unexpectedly, not the same fiber types being equipped with high activity of canonical Wnt signaling were compromised in conditional Lrp5 or Lrp6 knockout muscle fibers. Previously, low but detectable canonical Wnt signaling activity was analyzed in soleus muscles in comparison with other hind limb muscles where significantly more canonical Wnt signaling activity was detectable [22]. First, the number of slow type I fibers increased in conditional Lrp6, but not in Lrp5 knockout mice (Figure 3A,B). Second, the number of fast type IIa fibers decreased in conditional Lrp6, but not in Lrp5 knockout mice (Figure 3A,B). Third, cross-sectional areas of slow type I fibers were significantly lower in conditional Lrp6, but not changed in conditional Lrp5 knockout mice (Figure 3A,C). Fourth, cross-sectional areas of type IIa fibers were reduced in both, conditional Lrp5 and Lrp6, knockout mice (Figure 3A,C). Altogether, these data demonstrate changes of muscle fiber type distribution and cross-sectional areas, mainly for conditional Lrp6 knockout mice.

### 3.4. The Absence of Lrp5 or Lrp6 in Skeletal Muscle Fibers of Conditional Knockout Mice Determines the Differential Expression of Myogenic and Synaptic Markers

**Figure 4:**
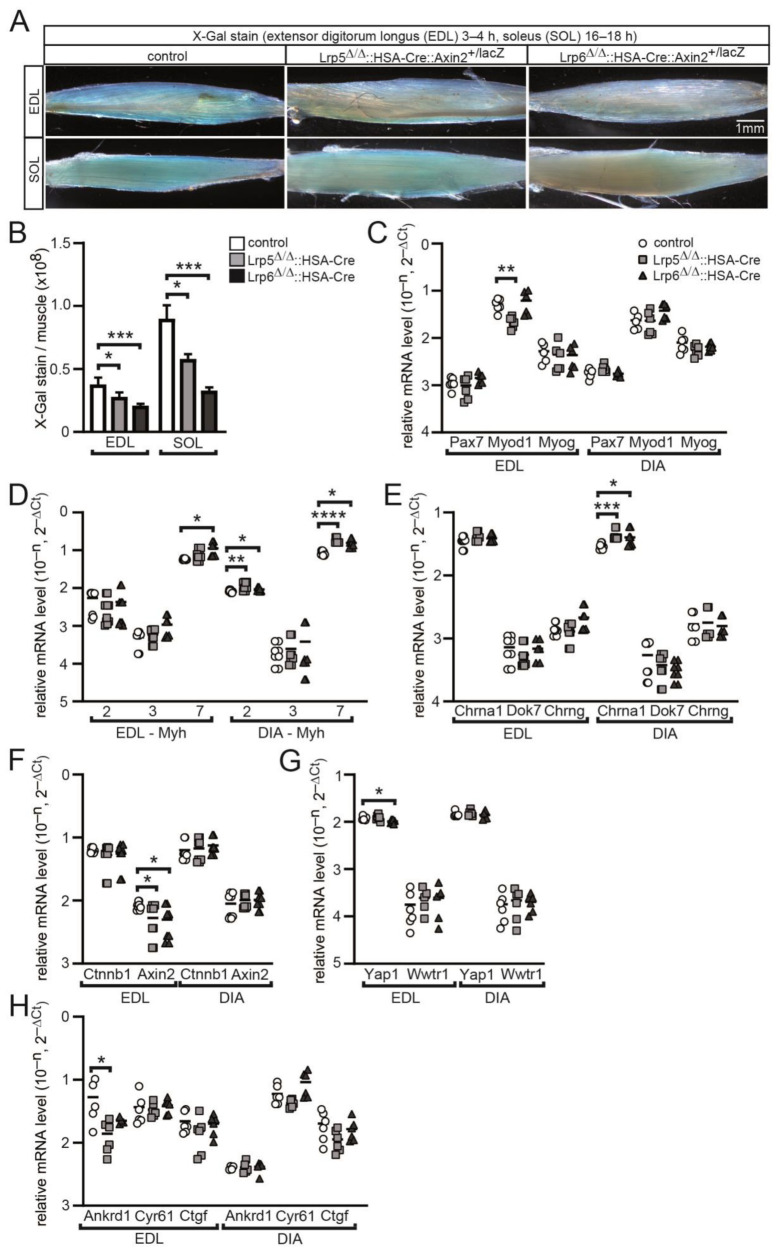
Altered transcription profile of myogenic genes in conditional Lrp5 or Lrp6 knockout mice. (**A**) Extensor digitorum longus (EDL) and soleus (SOL) muscles of control and different mutant mice (Lrp5^Δ/Δ^::HSA-Cre::Axin2^+/lacZ^ or Lrp6^Δ/Δ^::HSA-Cre::Axin2^+/lacZ^) were X-Gal-stained and whole mount color-imaged. (**B**) After color deconvolution, arbitrary values of total blue stain were detected by Fiji, and are presented by the graph. Note that the blue reporter stain mirrors active canonical Wnt signaling and is gradually decreased in conditional Lrp5 or Lrp6 knockout mice. Total RNA was extracted from EDL and diaphragm (DIA), 1st cDNA generated, and qPCR experiment performed. Graphs (bars and scatter plots) represent results for different sets of genes, (**C**) myogenic markers Pax7, Myod, Myog, (**D**) differentiation markers Myh2, Myh3, Myh7, (**E**) NMJ markers Chrna1, Chrng, Dok7, (**F**) Wnt signaling pathway markers Ctnnb1, Axin2, (**G**) Hippo transcriptional co-activators, Yap1, Wwtr1, (**H**) typical Hippo pathway targets Ankrd1, Cyr61, Ctgf. All labeling of graph bars and symbols in scatter graphs represents genotype of mice as indicated in (**B**,**C**). **** *p* < 0.0001, *** *p* < 0.001, ** *p* < 0.01, * *p* < 0.05.

Canonical Wnt signaling is employing transcriptional co-activator β-catenin to recruit members of the TCF/LEF transcription factor family for target gene expression. One such target gene is AXIN2. We wondered how gene expression might be modulated in absence of the canonical Wnt signaling co-receptors Lrp5 or Lrp6. We decided to make use of a previously published canonical Wnt signaling reporter mouse model, Axin2-LacZ [18,22]. By breeding, the Axin2-lacZ reporter was combined with conditional Lrp5 or Lrp6 knockout in adult mice and skeletal muscles of these mice were compared with control mice. Canonical Wnt signaling activity was monitored by blue-colored staining of adult muscle fibers in the extensor digitorum longus and soleus muscles of the mice (Figure 4A), a strategy which was described previously [22]. Employing color deconvolution to quantify the degree of blue stain, indeed, blue-stained adult muscle fibers appeared gradually less-colored in conditional Lrp5 and Lrp6 knockout mice in comparison with controls (Figure 4B). Next, we examined different regulation of several myogenesis and synaptic genes in conditional Lrp5 or Lrp6 knockout mice. Total RNA was extracted from diaphragm and extensor digitorum longus muscles of relevant mice and used for qPCR experiments. First, we analyzed transcript amounts of typical common myogenic markers such as Pax7, Myod1 and Myog. We did not detect any gross change of transcript amount for these three markers; Myod1 was a little decreased in extensor digitorum longus from conditional Lrp5 knockout mice (Figure 4C). Second, we looked for gene expression levels of differentiated myofiber markers, such as myosin heavy chain genes Myh2, Myh3 and Myh7 (Figure 4D). Interestingly, we detected several modulations regarding transcript amounts of these markers; prominently, Myh7 was changed in both muscles in conditional Lrp6 knockout mice (Figure 4D). Third, we analyzed synaptic genes Chrna1, Chrng, and Dok7 (Figure 4E). While Chrng was not changed in the mutants, in comparison to control, Chrna1 transcript amount was upregulated in both conditional Lrp knockout muscles (Figure 4E). Fourth, by investigating transcript amounts of signaling path members we determined that less transcript amount of a typical target gene of canonical Wnt signaling, Axin2, was detected (Figure 4F). The transcript level of Ctnnb1 was not different in both muscles of conditional Lrp5 and Lrp6 knockout mice in comparison to controls (Figure 4F). Transcript amounts of Hippo path members Yap1 and Wwtr1 were not much different; a little reduction of Yap1 transcript amount was detected in extensor digitorum longus muscles of conditional Lrp6 knockout mice (Figure 4G). Transcript amounts of typical Hippo target genes were mostly not changed; Ankrd1 was a little reduced in extensor digitorum longus muscles of conditional Lrp6 knockout mice (Figure 4H). In summary, myogenic and synaptic transcriptome of conditional Lrp5 or Lrp6 knockout muscles appeared to be modulated, but partly differently regulated between conditional Lrp5 or Lrp6 knockout mice.

### 3.5. The Endplate Band Widths of Conditional Lrp5 and Lrp6 Knockout Mice Are Enlarged and the Neuromuscular Junctions of Conditional Lrp6 Knockout Mice Are Fragmented and Accompanied by Impaired Neuromuscular Transmission

**Figure 5:**
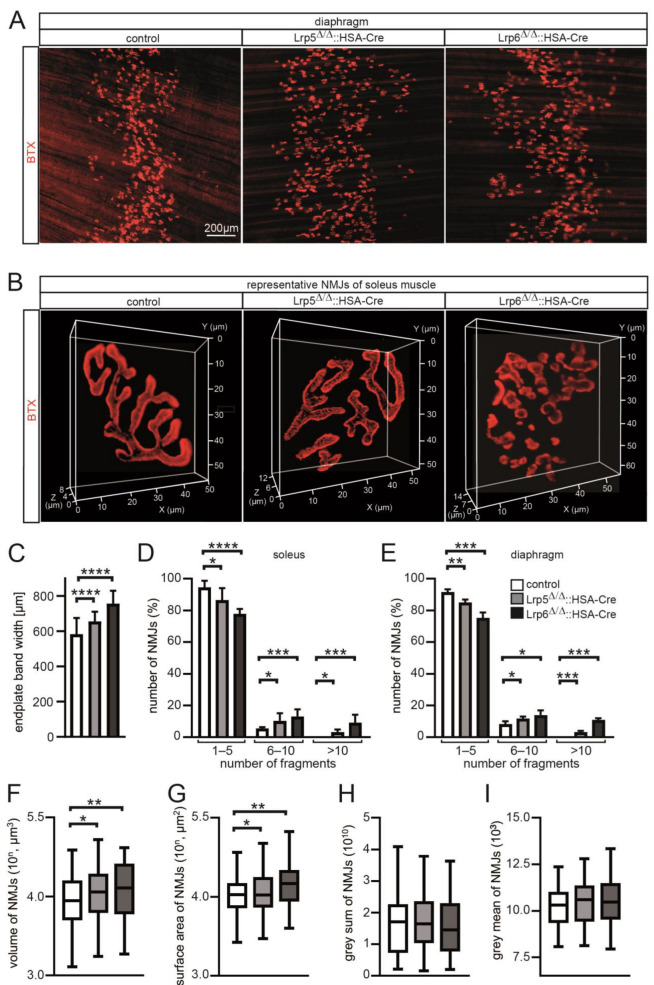
Endplate band width is gradually increased and NMJs are fragmented in conditional Lrp5 and Lrp6 knockout mice. (**A**) Representative images of BTX-stained diaphragms from control and mutant adult mice are shown. (**B**) Typical 3D images from individual NMJs of control and mutant soleus muscle fibers are presented. (**C**) Graph summarized endplate band width measured using diaphragm muscles of indicated mice. Note that endplate band width gradually increases in conditional Lrp5 or Lrp6 knockout mice in comparison with controls. (**D**,**E**) NMJ fragmentation grade is presented for soleus and diaphragm muscle fibers by a graph subgrouping three different ranges of NMJ fragment numbers. (**F**–**I**) Graphs summarize quantitative 3D imaging of NMJs of soleus muscle fibers of conditional Lrp5 or Lrp6 knockout mice in comparison with controls. Note that NMJ volume and surface area are changed in conditional Lrp5 or Lrp6 knockout mice in different degree in comparison with control mice. All labeling of graph bars represents genotype of mice as indicated in (**E**). **** *p* < 0.0001, *** *p* < 0.001, ** *p* < 0.01, * *p* < 0.05.

Active canonical Wnt signaling is believed to play a role at NMJs [22]. Hence, we looked for NMJs of conditional Lrp5 or Lrp6 knockout mice in comparison with controls. We dissected diaphragm and soleus muscles and stained them with BTX (Figure 5A,B). Endplate band width in conditional Lrp5 and Lrp6 knockout mice gradually increased in comparison with controls (Figure 5C). After 3D imaging of individual NMJs of soleus fibers, we detected several structural changes (Figure 5B,D,F–I). We quantified individual BTX-stained NMJ 3D images from dissected soleus fibers in an automated high-throughput fashion. We started by looking for NMJ fragmentation which is believed to represent the quality of NMJs, because pathologies or aging are known to increase the number of fragments that NMJs are composed of [28]. In comparison with controls, soleus of muscle fibers of conditional Lrp5 or Lrp6 knockout mice, and also diaphragm muscle fibers harbored fragmented NMJs (Figure 5B,D,E). NMJs of conditional Lrp6 knockout mice were more fragmented than those of conditional Lrp5 knockout mice (Figure 5B,D,E). Quantitative analysis of the 3D imaging data revealed changes in volume and surface area of BTX-stained NMJs of conditional Lrp6 knockout mice in comparison with controls (Figure 5F,G). While the percentage of NMJs composed of less than five fragments decreased gradually in conditional Lrp5- or Lrp6-deficient soleus muscle fiber bundles in comparison with controls, the percentage of NMJs with six to ten fragments significantly increased in conditional Lrp5, and even more in conditional Lrp6 knockout mice (Figure 5D). This correlation was also evident by quantitative 3D analysis of NMJ volume and surface area (Figure 5F,G). Total fluorescence intensities and mean fluorescence per pixel were not changed (Figure 5H,I), arguing for a lack of difference comparing total amount of AChRs or amount of AChR per pixel between control and mutant soleus NMJs. Of note, these structural data confirm different consequences of phenotypes in muscles of conditional Lrp5 and Lrp6 knockout mice.

**Figure 6:**
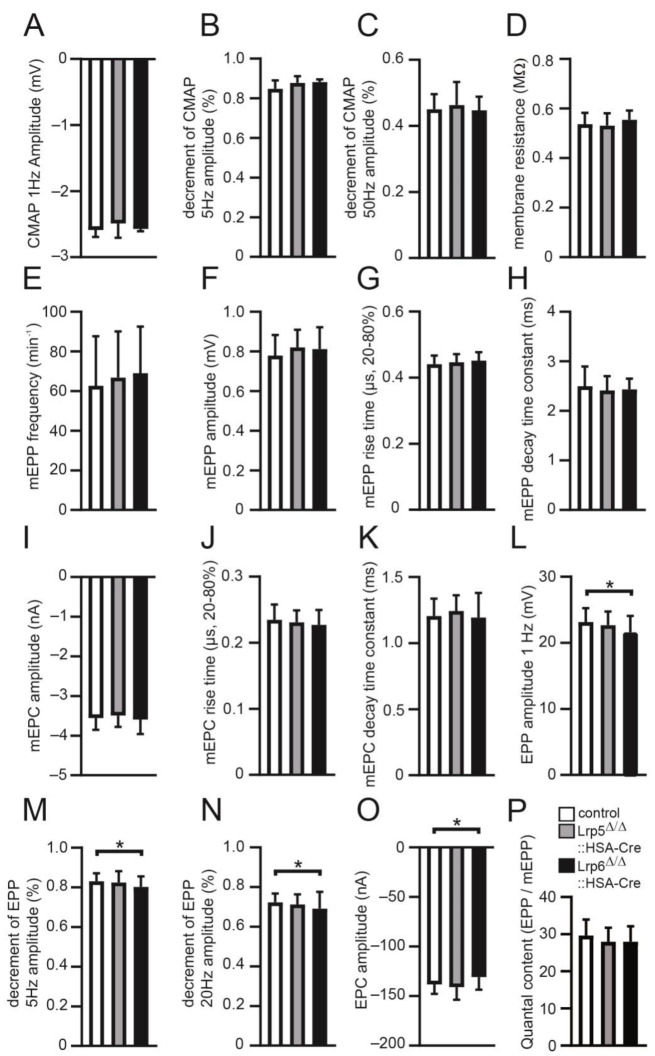
Characterization of neural transmission using conditional Lrp5 or Lrp6 knockout mice. Diaphragms of adult mice of indicated genotypes were used for electrophysiological recordings. Graphs present the analysis of the following parameters: (**A**) compound muscle action potential (CMAP) amplitude at 1 Hz, (**B**) decrement of CMAP amplitude at 5 Hz, (**C**) decrement of CMAP amplitude at 50 Hz, (**D**) membrane resistance, (**E**) miniature endplate potential (mEPP) frequency, (**F**) mEPP amplitude, (**G**) mEPP rise time, (**H**) mEPP decay time constant, (**I**) miniature endplate current (mEPC) amplitude, (**J**) mEPC rise time, (**K**) mEPC decay time constant, (**L**) endplate potential (EPP) amplitude at 1 Hz, (**M**) decrement of EPP amplitude at 5 Hz, (**N**) EPP amplitude at 20 Hz, (**O**) Endplate current (EPC) amplitude, (**P**) Quantal content (EPP/mEPP). Note that only recordings in conditional Lrp6 knockout diaphragms detect slight reductions in EPP amplitude at 1 Hz and the decrement in EPP amplitudes at 5 and 20 Hz. Data are represented as mean ± SD; *n* ≥ 5 mice per genotype and analysis of >20 individual NMJs per muscle. All labeling of graph bars represents genotype of mice as indicated in (**P**). * *p* < 0.05.

We continued addressing for any correlations of structure to function in conditional Lrp5 or Lrp6 knockout mice. To this end, we recorded extra- and intra-cellular potentials and currents in muscles of mutant mice in comparison with controls to analyze the physiology of neuromuscular transmission at their NMJs (Figure 6). By recording CMAPs, compound muscle action potentials that are triggered by consecutive nerve stimuli, we neither detected a difference between control and mutant mice with CMAP amplitude (Figure 6A), nor any significant change of the decrement of amplitudes at 5 and 50 Hz (Figure 6B,C). Membrane resistance values were comparable between different genotypes arguing for non-affected membrane integrity (Figure 6D). Moreover, recording of miniature endplate potentials (mEPP) did not reveal a significant change of the frequency (Figure 6E). Neither mEPP or miniature endplate current (mEPC) amplitudes were different in mutant mice arguing for unaffected local depolarizations around endplates in response to spontaneous acetylcholine release (Figure 6F,I), nor mEPP and mEPC rise time and decay time constants were changed in either mutant in comparison to controls (Figure 6G,H,J,K). On the other hand, EPP and EPC amplitudes, local responses at NMJs to nerve stimulation, were decreased in conditional Lrp6 knockout mice (Figure 6L,O). Run down experiments at 5 and 20 Hz demonstrated a decrease in EPP decrement for conditional Lrp6 knockout mice (Figure 6M,N). Quantal content, the mean number of quanta that are released to generate an EPP, was not changed in conditional Lrp knockout mice in comparison with controls (Figure 6P). In sum, no changes in neural transmission were recorded for conditional Lrp5 knockout mice. Altogether, our data demonstrate structural or functional impairments at the NMJs of adult conditional Lrp5 or Lrp6 knockout mice. Interestingly, impairments are more prominent in conditional Lrp6 knockout mice and less strong in conditional Lrp5 knockout mice.

## 4. Discussion

Active canonical Wnt signaling plays a key role in different aspects of skeletal muscle biology. While there is some knowledge available regarding the role of canonical Wnt signaling in early and late myogenesis and active signaling being present at NMJs [22], almost nothing is known regarding the need for the co-receptors of canonical Wnt signaling, Lrp5 and Lrp6, in skeletal muscles. Here, we used conditional Lrp5 or Lrp6 knockout mice to generate skeletal muscle-specific ablation of Lrp5 or Lrp6 expression by Cre recombinase mediated generation of knockout mice. We utilized HSA-Cre mice to ensure knockout of Lrp5 or Lrp6 at developmental stage, where myoblasts fuse to myotubes. With these mice, we investigated the role of Lrp5 and/or Lrp6 in adult muscle fibers or at NMJs. We aimed to understand whether both co-receptors are required for canonical Wnt signaling in adult muscle fibers and at NMJs, and whether we detect any specific changes distinguishing conditional Lrp5 from Lrp6 knockout mice. Previously, a different WNT3A response of Lrp5 or Lrp6 was found in osteoblasts arguing for a more important role of Lrp6 in comparison with Lrp5 [62]. Regarding the role of Lrp5 and/or Lrp6 at NMJs, this is even more exciting because an important regulator of NMJ formation and maintenance, the receptor tyrosine kinase MuSK, was reported to bind canonical Wnt ligands by its CRD and thereby act by itself like a canonical Wnt signaling receptor [35,63]. In patients, evidence accumulated for the pathophysiological importance of the MuSK CRD domain either by being linked to mutations in congenital myasthenic syndromes or by detection of autoantibodies in myasthenia gravis [64,65]. For better understanding, a mouse model lacking the CRD of MuSK was generated and characterized [63]. Interestingly, profound NMJ defects were detected in those mice at embryonic and adult stage culminating in muscle weakness [63]. The effects were reversible using lithium chloride, which is an agonist of the canonical Wnt signaling that can inhibit GSK3β activity and thereby stabilize free cytosolic β-catenin [63]. Obviously, it is interesting to understand the ways in which different canonical Wnt receptors are related to each other and whether the classical Wnt co-receptors, Lrp5 and Lrp6, are involved in canonical Wnt signaling at adult NMJs with or without MuSK CRD domain.

It is obvious to inquire the phenotype of the conditional Lrp5/Lrp6 double knockout mice. As mentioned above, the human skeletal actin promoter was reported to be also ectopically expressed [60]. We assume that ectopic Cre expression is the cause of lethality of conditional Lrp5/Lrp6 double knockout at embryonic stage ~E13 (Figure 1E). The use of other skeletal muscle Cre mice is required for further studies to completely understand the role of canonical Wnt signaling in muscle cells. Alternatively, the extraction of primary muscle satellite cells from mice expressing tamoxifen-inducible Cre recombinase might permit to establish Lrp5/Lrp6 double knockout muscle cells in vitro. Differentiation of such mononuclear myoblasts to multi-nucleated myotubes might allow to monitor the consequences of double knockout muscle cells regarding proliferation, myotube formation, and clustering of AChR upon incubation of myotubes with AGRIN-conditioned cell culture media. These possibilities for investigating the phenotype of the double Lrp5/Lrp6 knockout muscle cells or mice should be considered in future studies.

Here, our data evidence the importance of Lrp5 and Lrp6 acting together, because double knockout mice are lethal before birth, while single knockouts mice are viable (Figure 1E). This means that the ectopic expression and subsequent knockout of one of the Lrp genes has a less serious effect than the knockout of both Lrp genes in those ectopic cells, where HSA-Cre is expressed. Previous reports indicate a more severe phenotype in Lrp6 in comparison with Lrp5 knockout mice [66,67]. Interestingly, our data analyzing conditional Lrp5 or Lrp6 knockout muscles are in agreement with the previous data, arguing for non-related function of these two co-receptors. Both conditional Lrp5 or Lrp6 knockout mice show profound impairments in adult skeletal muscle fibers and at NMJs. In adult muscle fibers, (1) oxidative metabolism is little reduced in mutant mice in comparison with controls (Figure 2A,C,D), (2) myosin heavy chain distribution and cross-sectional areas are disorganized in conditional Lrp6 knockout mice (Figure 3), (3) canonical Wnt target gene expression is reduced (Figure 4A,B), and (4) myogenic gene expression profiles are partially modified (Figure 4C,D). At the NMJs of conditional Lrp5 or Lrp6 knockout mice, we observed (1) slightly altered synaptic gene expression (Figure 4E), (2) enlarged endplate width in diaphragm muscle (Figure 5A,C), (3) fragmentation of NMJs (Figure 5B,D,E), and (4) slightly but significantly impaired neuromuscular transmission (Figure 6). The data we obtained for adult muscle fibers in mutant mice suggest a potential involvement of canonical Wnt signaling in fiber type distribution and metabolic status of the fibers. At NMJs, it appears that canonical Wnt signaling is influenced by the classical Wnt co-receptors Lrp5 and Lrp6, and their role in relation to MuSK-CRD requires further study. Interestingly, endplate band width is gradually enlarged in conditional Lrp5 or Lrp6 knockout mice (Figure 5A,C). It might be worth to analyze whether prepatterning is differently affected in conditional Lrp5 or Lrp6 knockout mice by analyzing diaphragms of E13 and E18 embryos. Fragmentation of NMJs looking at conditional Lrp5 or Lrp6 knockout mice (Figure 5B,D,E) might be related to modified synaptic gene expression (Figure 4E).

Previously, conditional muscle-specific knockout of β-catenin, the key transcriptional co-activator of canonical Wnt signaling, was shown to consequence in a retrograde regulation of motoneuron differentiation in mice [29]. In our experiments, the gene expression level of β-catenin is unchanged in conditional Lrp5 or Lrp6 knockout mice (Figure 4F), but the expression level of a typical canonical Wnt target gene, Axin2, is significantly reduced (Figure 4A,B,F). It is likely that both Lrp5 and Lrp6 should be knocked out to detect retrograde regulation of motoneuron differentiation.

In summary, both Lrp5 and Lrp6 apparently play different roles in adult skeletal muscle fibers and at NMJs; they are not replaceable by each other. Future studies also involving the double knockout and the MuSK CRD mutant mice should help to address many more questions and move towards better understanding of the role of WNT receptors in skeletal muscle biology.

## Supplementary Materials

The following supporting information can be downloaded at: https://www.mdpi.com/article/10.3390/cells11243968/s1, Table S1: Tabular presentation of oligonucleotide sequences.
