# Supplementary material for: In Adult Skeletal Muscles, the Co-Receptors of Canonical Wnt Signaling, Lrp5 and Lrp6, Determine the Distribution and Size of Fiber Types, and Structure and Function of Neuromuscular Junctions
Source: Cells. 2022 Dec 8;11(24):3968. doi: 10.3390/cells11243968 (PMC9777411; doi:10.3390/cells11243968)
Supplement: Supplementary file 1 [file cells-11-03968-s001.zip › cells-2023963-supplementary.pdf]

SUPPLEMENTARY DATA

Supplementary Table S1

Tabular presentation of oligonucleotide sequences

Genomic target	Orientation	Sequence
Dok7 qPCR	forward	GAATTCGGTTCTCTGCTCAGTCTG
	reverse	CCAAGTCCATGTAGTGCAGCTG
Chrna1 qPCR	forward	ACGCTGAGCATCTCTGTCTT
	reverse	TTGGACTCCTGGTCTGACTT
Chrng qPCR	forward	GGTCAATGTCAGCCTGAAGC
	reverse	GCACATGCATCCGTAACAGC
Yap1 qPCR	forward	TTCGGCAGGCAATACGGAAT
	reverse	GTTGAGGAAGTCGTCTGGGG
Wwtr1 qPCR	forward	GTTCCGGGGATAAAGATGAATCCG
	reverse	GAAGTGATGGACGGGTGGAG
Rpl8 qPCR	forward	GTTCGTGTACTGCGGCAAGA
	reverse	ACAGGATTCATGGCCACACC
Cyr61 qPCR	forward	AAGAGGCTTCCTGTCTTTGGC
	reverse	ATCGGAACCGCATCTTCACA
CTGF qPCR	forward	CTAGCTGCCTACCGACTGGAA
	reverse	CAAACTTGACAGGCTTGGCG
Ankrd1 qPCR	forward	TGGAGGAAACGCAGATGTCC
	reverse	TCCCAGCACAGTTCTTGACC
Axin2 qPCR	forward	GACGGACAGTAGCGTAGATGG
	reverse	GGGTCCTCTTCATAGCTGCC
Pax7 qPCR	forward	GCTACCAGTACAGCCAGTATG
	reverse	GTCACTAAGCATGGGTAGATG
Myod1 qPCR	forward	TGGCATGATGGATTACAGCGG
	reverse	GGTCTGGGTTCCCTGTTCTG
Myog qPCR	forward	CAGTACATTGAGCGCCTACA
	reverse	GCCTGACAGACAATCTCAGT
Myh2 qPCR	forward	AGAGTCCCGAACGAGGCTGACTC
	reverse	TCAGGGTCGCTCCTGCTTCTGTT
Myh3 qPCR	forward	TCCAAACCGTCTCTGCACTGTT
	reverse	AGCGTACAAAGTGTGGGTGTGT
Myh7 qPCR	forward	CTCAAGCTGCTCAGCAATCTATTT
	reverse	GGAGCGCAAGTTTGTCATAAGT
Ctnnb1 qPCR	forward	TCTGGAATCCATTCTGGTGC
	reverse	CTCATCTAGCGTCTCAGGGA
